# Role of ZNF224 in c-Myc repression and imatinib responsiveness in chronic myeloid leukemia

**DOI:** 10.18632/oncotarget.23283

**Published:** 2017-12-15

**Authors:** Gaetano Sodaro, Elena Cesaro, Giorgia Montano, Giancarlo Blasio, Federica Fiorentino, Simona Romano, Arnaud Jacquel, Patrick Aurberger, Paola Costanzo

**Affiliations:** ^1^ Department of Molecular Medicine and Medical Biotechnology, University of Naples, Federico II, Naples 80131, Italy; ^2^ Department of Haematology and Transfusion Medicine, BioMedical Center, Lund University, Lund 22184, Sweden; ^3^ Université Côte d’Azur, Inserm, Nice 06204, France

**Keywords:** ZNF224, chronic myeloid leukemia, c-Myc, imatinib, AG490

## Abstract

The transcription factor ZNF224 plays a key proapoptotic role in chronic myelogenous leukemia (CML), by modulating Wilms Tumor protein 1 (WT1) dependent apoptotic genes transcription. Recently, we demonstrated that Bcr-Abl signaling represses ZNF224 expression in Bcr-Abl positive CML cell lines and in CML patients. Interestingly, Imatinib and second-generation tyrosine kinase inhibitors specifically increase ZNF224 expression.

On the other hand, Bcr-Abl positively modulates, via JAK2 activation, the expression of the c-Myc oncogene, which is required for Bcr-Abl oncogenic transformation in CML. Consequently, JAK2 inhibitors represent promising molecular therapeutic tools in CML.

In this work, we demonstrate that ZNF224 is a novel transcriptional repressor of c-Myc in CML. We also show that ZNF224 induction by Imatinib and AG490, a specific JAK2 inhibitor, is responsible for the transcriptional repression of c-MYC, thus highlighting the crucial role of the ZNF224/c-Myc axis in Imatinib responsiveness.

Interestingly, we also report that ZNF224 is induced by AG490 in Imatinib-resistant CML cells, leading to c-Myc repression and apoptosis induction. These findings suggest that the development of molecular tools able to induce ZNF224 expression could provide promising means to bypass Imatinib resistance in CML.

## INTRODUCTION

The chimeric Bcr-Abl fusion oncoprotein is a product of a reciprocal chromosomal translocation between the long arms of chromosomes 9 and 22 t(9;22)(q34;q11) [1] and exerts a critical role in chronic myelogenous leukemia (CML) pathogenesis [2–4]. Constitutive tyrosine kinase activity of Bcr-Abl causes the activation of a multitude of signaling pathways, including Jak/STAT [5], PI3K/Akt [6, 7], Ras [8] and NF-kB [9], which eventually lead to the induction of several oncogenic transcription factors, important for sustaining cellular transformation in CML.

c-Myc is one of the oncogenic transcription factors induced by Bcr-Abl. It plays a central role in the regulation of proliferation, differentiation, apoptosis and tumorigenesis of hematopoietic cells and is necessary for Bcr-Abl oncogenic transformation in CML [10]. Consistently, the inhibition of Bcr-Abl tyrosine kinase activity by Imatinib strongly reduces c-Myc expression and hematopoietic tumoral features of CML cells. Importantly, c-Myc reduction represents a key step for Imatinib induced cell death in CML; indeed, elevated c-Myc levels are found during CML blast crisis phase and correlate with poor response to Imatinib [11–13].

The induction of c-Myc by Bcr-Abl occurs mainly via JAK2 activation, which positively regulates c-Myc protein and mRNA levels, even though the mechanisms involved in c-Myc transcriptional regulation are still largely unknown [14–17].

In agreement with the JAK2 role in mediating Bcr-Abl induction of c-Myc, JAK2 kinase inhibitors, such as AG490, strongly reduce c-Myc expression in CML and induce apoptosis [14, 16], thus indicating JAK2 pathway as an important therapeutic target to overcome Imatinib resistance in CML, a crucial issue in clinical practice [17–19].

The Kruppel-like zinc-finger protein ZNF224 is a transcriptional repressor, which has been recently proposed to play a dual role in carcinogenesis, acting as a tumor suppressor or an oncogene, depending on molecular partners and cellular contest [20, 21].

In CML, ZNF224 exerts a pro-apoptotic role, acting as a co-factor of the Wilms tumor protein 1 (WT1) transcription factor. ZNF224 is recruited by WT1 on the promoter of apoptosis-regulating genes and, modulating WT1 dependent transcription, it shifts the balance of antiapoptotic and proapoptotic signals in favor of the latter. Through this mechanism, ZNF224 plays a central role in ara-C-induced apoptosis in CML [22–24]. Moreover, we recently found that Bcr-Abl negatively regulates the expression of ZNF224 in CML cells via transcriptional repression; consistently, inhibition of Bcr-Abl tyrosine kinase activity, by Imatinib and second generation tyrosine kinase inhibitors, resulted in the up-regulation of ZNF224 expression [25].

In the present study, we demonstrate that ZNF224 represses c-Myc transcription in CML and coherently hampers c-Myc proliferative network, reducing CML cells proliferation and DNA synthesis. Importantly, we also demonstrate that ZNF224 mediates Imatinib and AG490 dependent down-modulation of c-Myc and apoptosis induction in K562 cells. Furthermore, we provide convincing evidence that ZNF224 induction by AG490 could play a role in overcoming Imatinib resistance in CML cells.

## RESULTS

### ZNF224 exerts a transcriptional repression on c-Myc expression

Our previous findings demonstrated that Bcr-Abl fusion protein negatively regulates the expression of the pro-apoptotic transcription factor ZNF224 in CML and accordingly Bcr-Abl inhibition by Imatinib and second-generation tyrosine kinase inhibitors (TKIs) Dasatinib and Nilotinib increase ZNF224 expression [25].

**Figure 1:**
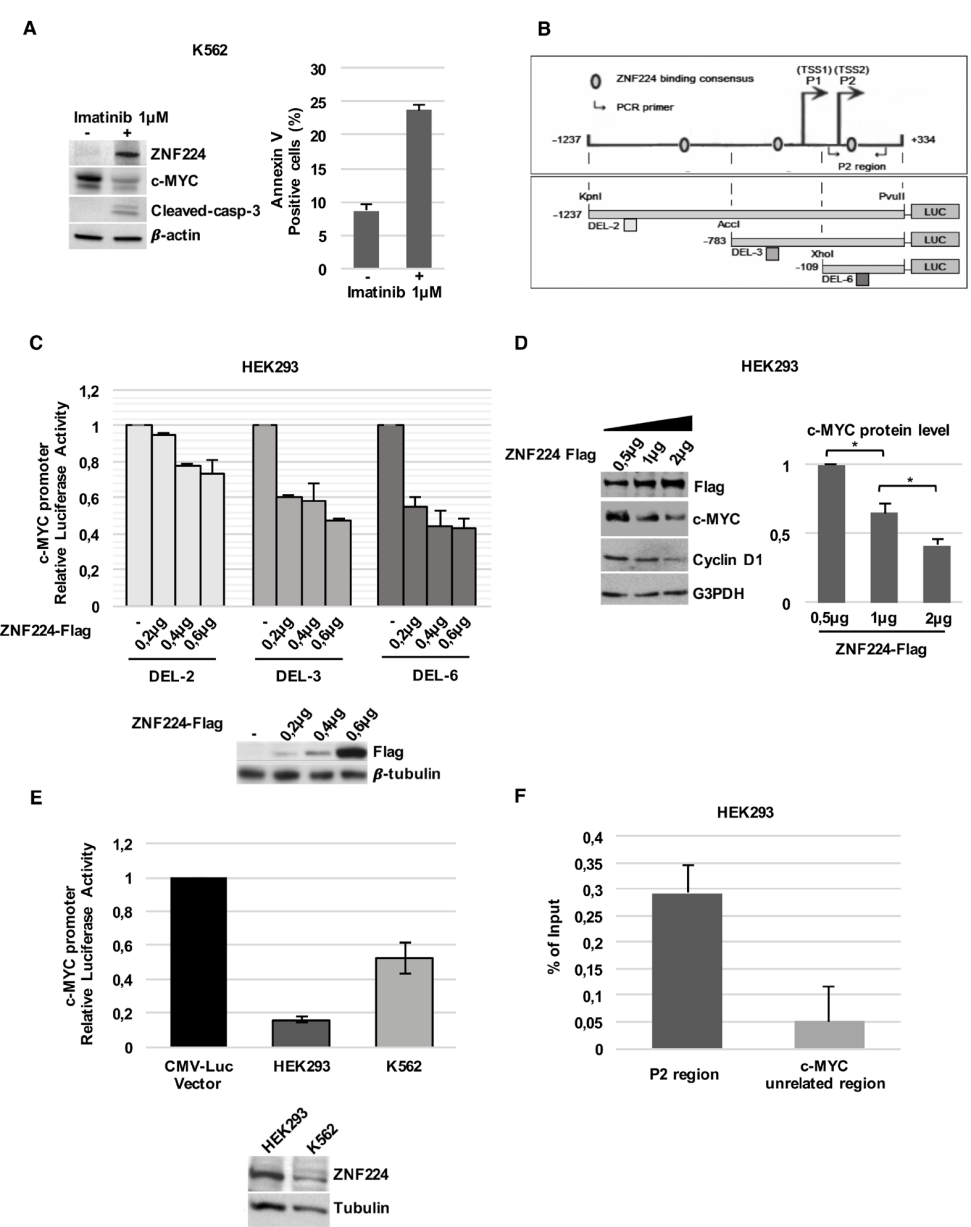
ZNF224 reduces c-Myc expression via a transcriptional mechanism (**A**) Western blot analysis of ZNF224, c-Myc and cleaved caspase-3 protein levels in K562 cells treated with Imatinib or vehicle only (DMSO), as control (−), for 48 hours . β-actin was used as loading control. One representative blot out of two performed is shown (left panel). Cell death was evaluated by annexinV-PE staining followed by flow cytometry. Results represent the means +/− SD of two independent experiments (right panel). (**B**) Schematic representation of c-Myc promoter region and DEL-2, DEL-3 and DEL-6 deletion constructs. (**C**) DEL-2, DEL-3 and DEL-6 constructs were transfected into HEK293 cells together with increasing amounts of 3X-Flag ZNF224 or 3X-Flag empty vector as control (−). After 24 h, the promoter activity was determined by normalizing Firefly to Renilla luciferase activity. Error bars represent standard deviations of two independent experiments. Expression of ZNF224-Flag was verified by western blot analysis. β-tubulin was used as loading control. One representative blot out of three performed is presented. (**D**) Western blot analysis of ZNF224-Flag, c-Myc and cyclin D1 protein levels in HEK293 cells transfected with increasing amounts of 3X-Flag ZNF224. G3PDH was used as loading control (left panel). Densitometric analysis of c-Myc protein levels. Error bars represent standard deviations of three independent experiments; ^*^p < 0.05 (right panel). (**E**) DEL-6 construct was transfected into HEK293 cells and K562 cell. After 24 h, promoter activity was determined by normalizing Firefly to Renilla luciferase activity. DEL-6 activity was compared to CMV Luciferase activity obtained in each cell line. Error bars represent standard deviations of two independent experiments. ZNF224 expression was measured by western blot analysis. β-tubulin was used as loading control. One representative blot out of two performed is shown. (**F**) ChIP assay performed with an anti-ZNF224 antibody. Quantitative RT-qPCR analysis was performed using primers flanking the P2 region. A region downstream c-Myc locus was used as negative control (c-Myc unrelated region). Error bars indicate the mean value +/− SD of two independent experiments.

The c-Myc oncogene is a target of Bcr-Abl protein transforming activity; indeed, Bcr-Abl inhibition by Imatinib strongly reduces c-Myc expression [10–13]. Coherently, we observed that ZNF224 induction by Imatinib well correlated with c-Myc downregulation and cell death induction in K562 CML cells (Figure 1A). Moreover, *in silico* analysis revealed the existence of three putative ZNF224 binding sites on the c-Myc promoter region, from nucleotides –1237 to +334, including the two transcriptions start sites (TSS) P1 and P2 (Figure 1B, upper panel). This suggests that c-Myc gene could be a target of ZNF224 transcriptional repression in CML.

To assess whether c-Myc promoter activity was affected by ZNF224 and to investigate the regions of c-Myc promoter involved in this regulation, we introduced three luciferase reporter plasmids containing progressive deletions of the c-Myc promoter (Figure 1B, lower panel) into HEK293 cells in the presence of increasing amounts of a ZNF224 expression vector; as shown in Figure 1C, c-Myc promoter transcriptional activity was progressively decreased by ZNF224 overexpression in all the three deletion mutants, thus indicating that ZNF224 represses c-Myc gene through the binding at the high regulatory P2 region of the c-Myc promoter, that is included in the DEL-6 construct.

In agreement with the results of luciferase assays, ZNF224 overexpression in HEK293 cells also reduced c-Myc protein levels in a dose-dependent manner and was associated with a decrease in cyclin D1 protein levels, a positive c-Myc target gene (Figure 1D). Consistently, we found that the basal transcriptional activity of DEL-6 construct was higher in K562 CML cells, which express lower levels of endogenous ZNF224, compared to HEK293 cells (Figure 1E).

To confirm ZNF224 binding on the P2 region of the c-Myc promoter, we conducted Chromatin immunoprecipitation assays (ChIP) in HEK293 cells. Chromatin was immunoprecipitated with a ZNF224 antibody and RT-qPCR analysis confirmed that ZNF224 was able to bind the P2 region of the c-Myc promoter (Figure 1F).

### ZNF224 binds to a regulatory element in the c-Myc promoter in CML

**Figure 2:**
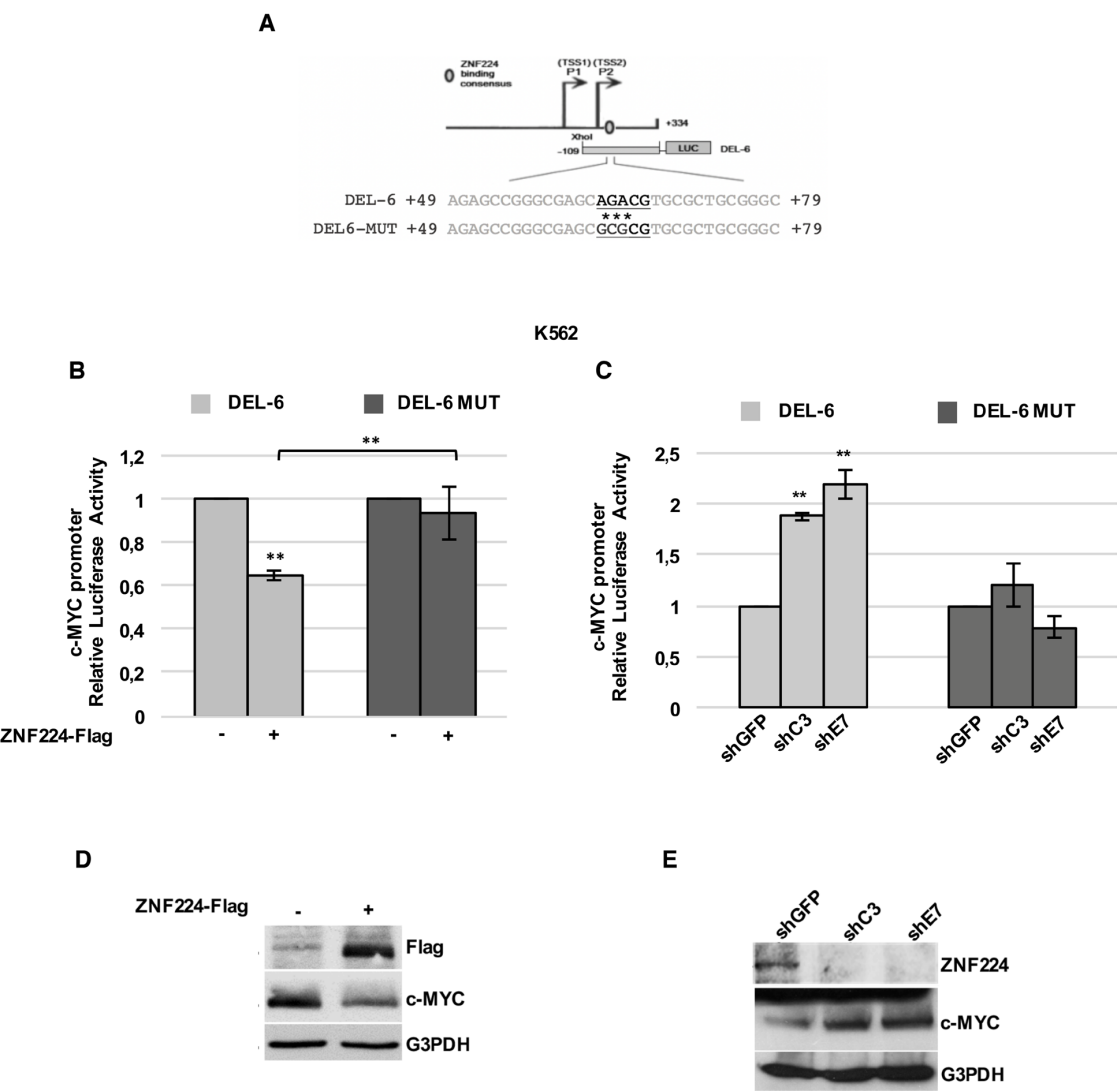
ZNF224 binding on P2 region is crucial for its repression activity on c-Myc promoter in CML (**A**) Schematic representation of DEL-6 and DEL-6 MUT constructs. Asterisks (*) indicate mutated bases. (**B**) K562 cells were transfected with DEL-6 or DEL-6 MUT together with 3X-Flag ZNF224 or 3X-Flag empty vector as control (−). After 48 h, promoter activity was determined by normalizing Firefly to renilla luciferase activity. Error bars represent standard deviations of three independent experiments; ^**^p < 0.005. (**C**) shC3, shE7 and shGFP cells were transfected with DEL-6 or DEL-6 MUT. After 24 h, the Firefly luciferase activity was measured and normalized to Renilla luciferase activity. Error bars represent standard deviations of three independent experiments; ^**^p < 0.005. (**D**) Western blot analysis of ZNF224-Flag and c-Myc protein levels in K562 transfected with 3X-Flag ZNF224 or 3X-Flag empty vector as control (−). G3PDH was used as loading control. (**E**) Western blot analysis of ZNF224 and c-Myc protein levels in shC3, shE7 and shGFP cells. G3PDH was used as loading control.

To confirm the transcriptional repression exerted by ZNF224 on c-Myc in K562 cells and to identify the ZNF224 binding element within the c-MYC promoter, we performed a site-directed mutagenesis of three nucleotides within the ZNF224 binding consensus on DEL-6 construct, obtaining the mutant DEL-6 MUT (Figure 2A), and compared the effect of ZNF224 on the transcriptional activity of wild-type and mutant c-Myc promoter. For this purpose, K562 cells were transfected with DEL-6 or DEL-6 MUT constructs together with the ZNF224-Flag expression vector and luciferase activity was measured. We observed that ZNF224 overexpression significantly reduced c-Myc DEL-6 promoter activity as expected, while it was not able to repress the luciferase activity of DEL-6 MUT construct (Figure 2B).

Furthermore, we transfected DEL-6 or DEL-6 MUT constructs in K562 cells stably knocked-down for ZNF224 (shC3 and shE7 cells) or in shGFP control cells. As shown in Figure 2C, DEL-6 promoter activity was increased by ZNF224 stable knockdown, while the luciferase activity of DEL-6 MUT was not affected by ZNF224 knockdown. Accordingly, we found a substantial reduction in c-Myc protein levels when ZNF224 was overexpressed (Figure 2D), while ZNF224 knocked-down cells showed increased c-Myc protein levels compared to control cells (Figure 2E).

As expected, we observed that the modulation of ZNF224 expression affects c-myc mRNA levels (Supplementary Figure 1).

Apart from its role in regulating cell death and survival, c-Myc oncogene plays a pivotal function in CML oncogenic transformation, mainly by increasing the cell proliferation rate [26–28].

**Figure 3:**
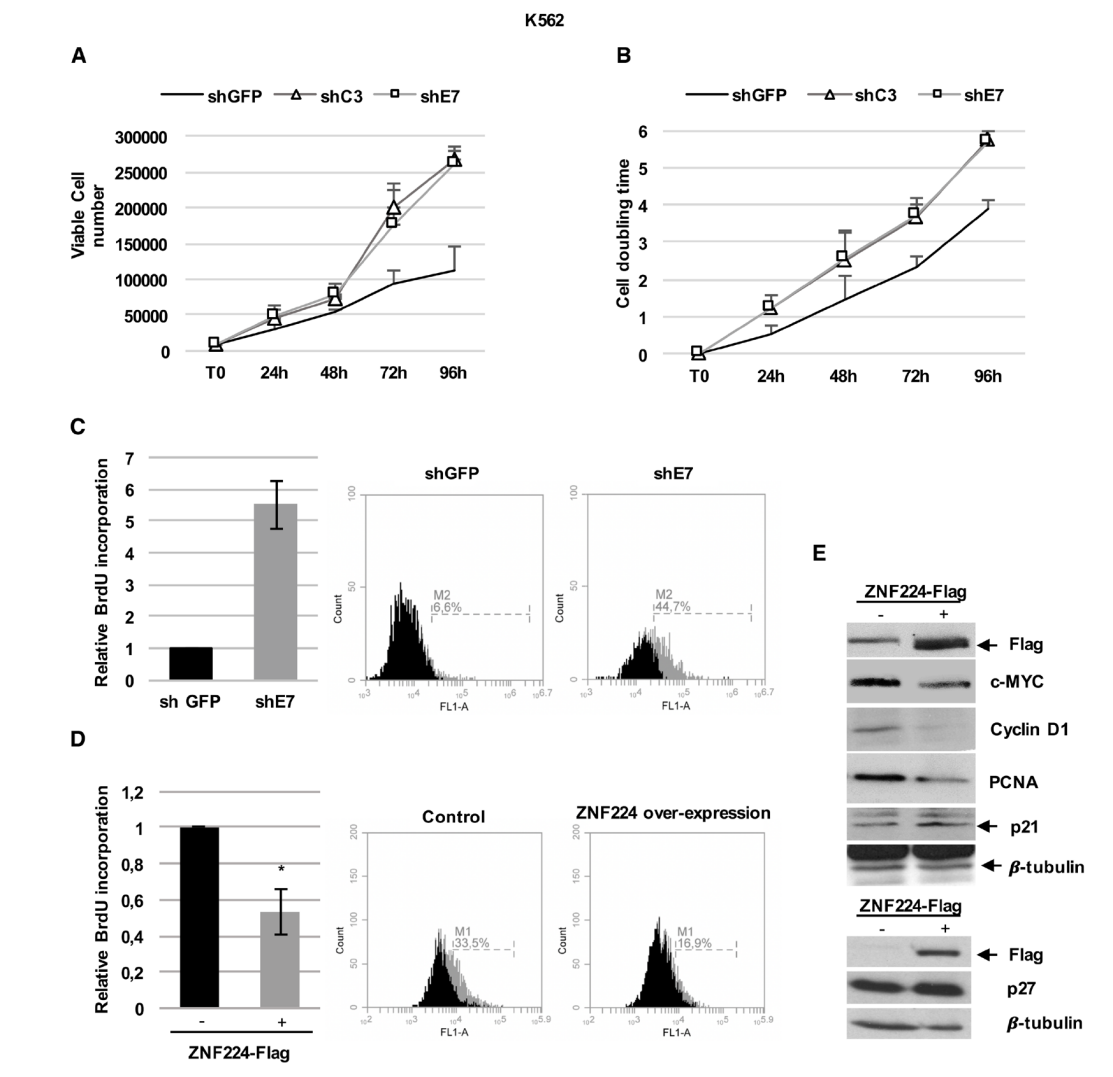
ZNF224 hampers CML cells proliferation (**A**) Proliferation rate of shC3, shE7 and shGFP cells evaluated by cell counting. Error bars represent standard deviations of three independent experiments. (**B**) Proliferation rate evaluated by cell doubling time. Error bars represent standard deviations of two independent experiments. (**C**) Relative BrdU incorporation in shE7 cells compared to shGFP cells. Error bars represent standard deviations of two independent experiments. Percentage of BrdU incorporation of one representative plot out of two is presented. (**D**) Relative BrdU incorporation in K562 cells transiently transfected with 3X-Flag ZNF224 expression vector compared to K562 cells transfected with 3X-Flag empty vector, used as control (−). Error bars represent standard deviations of three independent experiments.^*^p < 0.05. (**E**) ZNF224-Flag, c-Myc, cyclin D1, PCNA, p21 and p27 levels were analyzed by western blot. Arrows indicate specific bands. β-tubulin was used as loading control. One representative blot out of two performed is presented.

To further support our findings showing ZNF224 repression of c-Myc transcription in CML, we evaluated the proliferation rate in shC3 and shE7 cells. According to c-Myc increased levels, ZNF224 knockdown was associated with an increase in both K562 cell number (Figure 3A) and doubling time (Figure 3B). Furthermore, by BrdU incorporation assays we observed that ZNF224 knockdown was also accompanied by a significant increase in DNA synthesis (Figure 3C). Coherently, a significant decrease in DNA synthesis was observed when ZNF224 was overexpressed (Figure 3D). Finally, we observed that ZNF224 overexpression was accompanied by a decreased proliferative profile, with a reduction of c-Myc, cyclin D1 and PCNA and an increase of p21 and p27 protein levels (Figure 3E).

These data show that ZNF224 exerts a transcriptional repression on c-Myc expression in CML cells and coherently inhibits cell proliferation.

### ZNF224 mediates the Imatinib-dependent transcriptional repression of c-Myc in CML and induces cell death in Imatinib-resistant CML cells

Starting from these data, we decided to investigate whether ZNF224 was involved in Imatinib-mediated transcriptional repression on c-Myc oncogene, which represents a key event in Imatinib responsiveness in CML [11–13].

**Figure 4:**
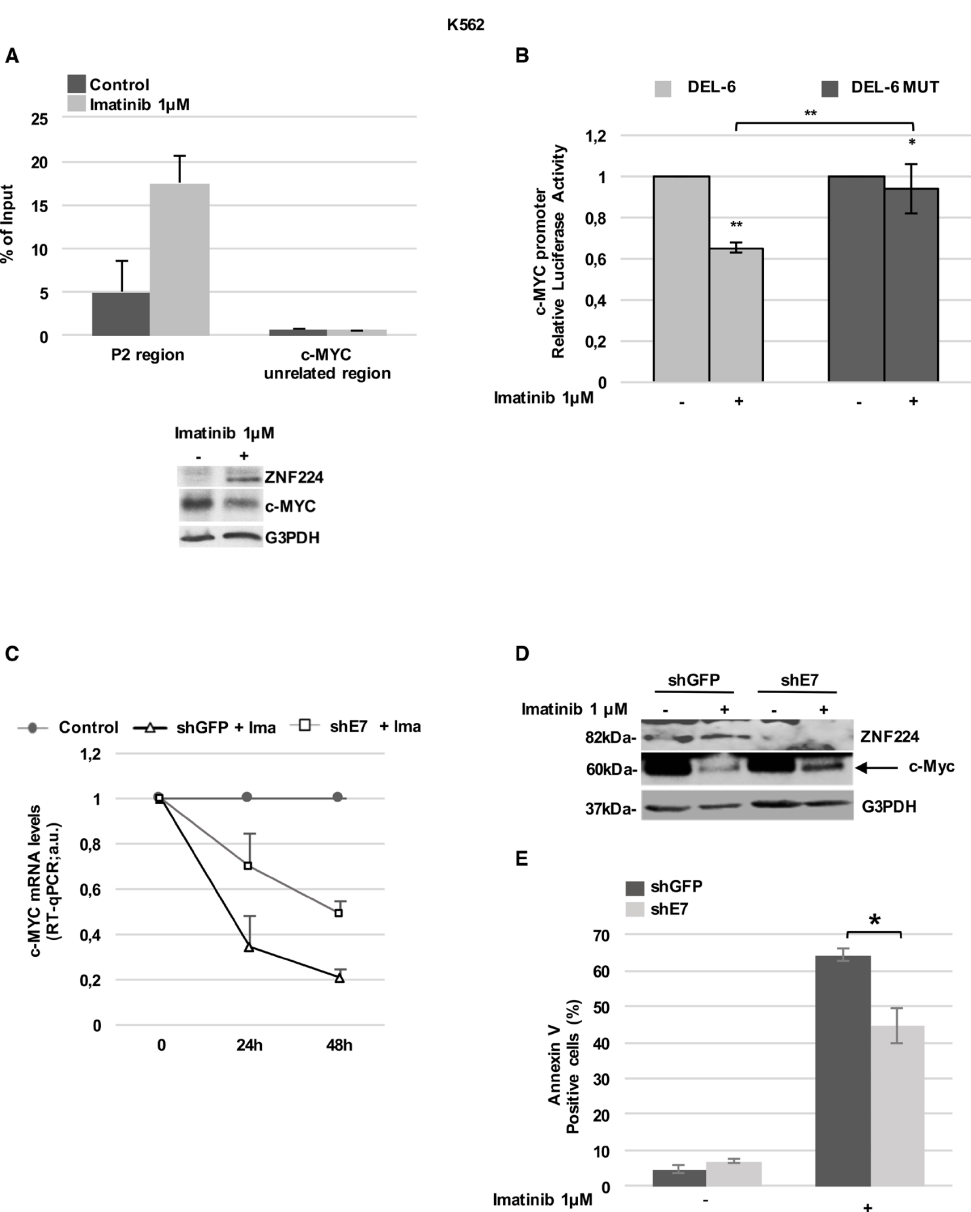
ZNF224 mediates the Imatinib-dependent transcriptional repression on c-Myc (**A**) ChIP assay performed with an anti-ZNF224 antibody in K562 cells treated with Imatinib or vehicle only (DMSO) as control for 24 h. Quantitative RT-qPCR analysis was performed using primers covering the P2 region. A c-Myc unrelated region was used as negative control. Error bars indicate the mean value +/− SD of two independent experiments (upper panel). ZNF224 and c-Myc protein levels were analyzed by western blot. G3PDH was used as loading control. One representative blot out of two performed is shown (lower panel). (**B**) K562 cells were transfected with DEL-6 or DEL-6-MUT constructs and after 24 hours treated with Imatinib. Luciferase activity was determined 24 hours later and promoter activity was normalized to Renilla luciferase activity. Error bars represent standard deviations of two independent experiments. ^*^p < 0.05; ^**^p < 0.005. (**C**) shE7 and shGFP cells were exposed to Imatinib or vehicle only (DMSO) for 48 hours. c-Myc mRNA levels were measured by RT-qPCR. Relative amounts of c-Myc mRNA levels in shE7 and shGFP cells treated with Imatinib were compared to those in shE7 and shGFP cells treated with DMSO (control). Error bars represent standard deviations of two independent experiments. (**D**) ZNF224 and c-Myc protein levels were measured by western blot analysis. Arrow indicates specific band. G3PDH was used as loading control. One representative blot out of two is presented. (**E**) Cell death was determined by annexin V staining followed by flow cytometry. Error bars represent standard deviations of three independent experiments. ^*^p < 0.05.

We firstly examined ZNF224 occupancy on the c-Myc promoter in K562 cells and the effect of Imatinib treatment on this binding by ChIP assays. To this aim, K562 cells were incubated in the absence or presence of Imatinib 1µM for 24h, then chromatin was immunoprecipitated with a ZNF224 antibody. As shown in Figure 4A, real-time qPCR of immunoprecipitated chromatin revealed a basal ZNF224 occupancy on the P2 region of the c-Myc promoter that was considerably increased by Imatinib treatment.

Then, we investigated the effect of Imatinib on the activity of the c-Myc promoter constructs DEL-6 and DEL-6 MUT. As shown in Figure 4B, Imatinib treatment reduced DEL-6 promoter activity, while a significantly lower repression was observed on the DEL-6 MUT.

In agreement with ZNF224 induction by Imatinib, these results suggest that ZNF224 binding on c-Myc promoter is required for Imatinib repression of the c-Myc gene.

To further confirm the ZNF224 role in Imatinib-dependent repression of c-Myc, shE7 cells were treated with 1 µM Imatinib for 48 hours, after which c-Myc expression was evaluated. Interestingly, we found that ZNF224 knockdown impaired Imatinib-dependent downregulation of c-Myc mRNA (Figure 4C) and protein levels (Figure 4D). In addition, as expected, ZNF224 knockdown significantly reduced the cell death induced by Imatinib (Figure 4E). Collectively, these results highlight a mechanism by which ZNF224 contributes to Imatinib responsiveness in CML.

**Figure 5:**
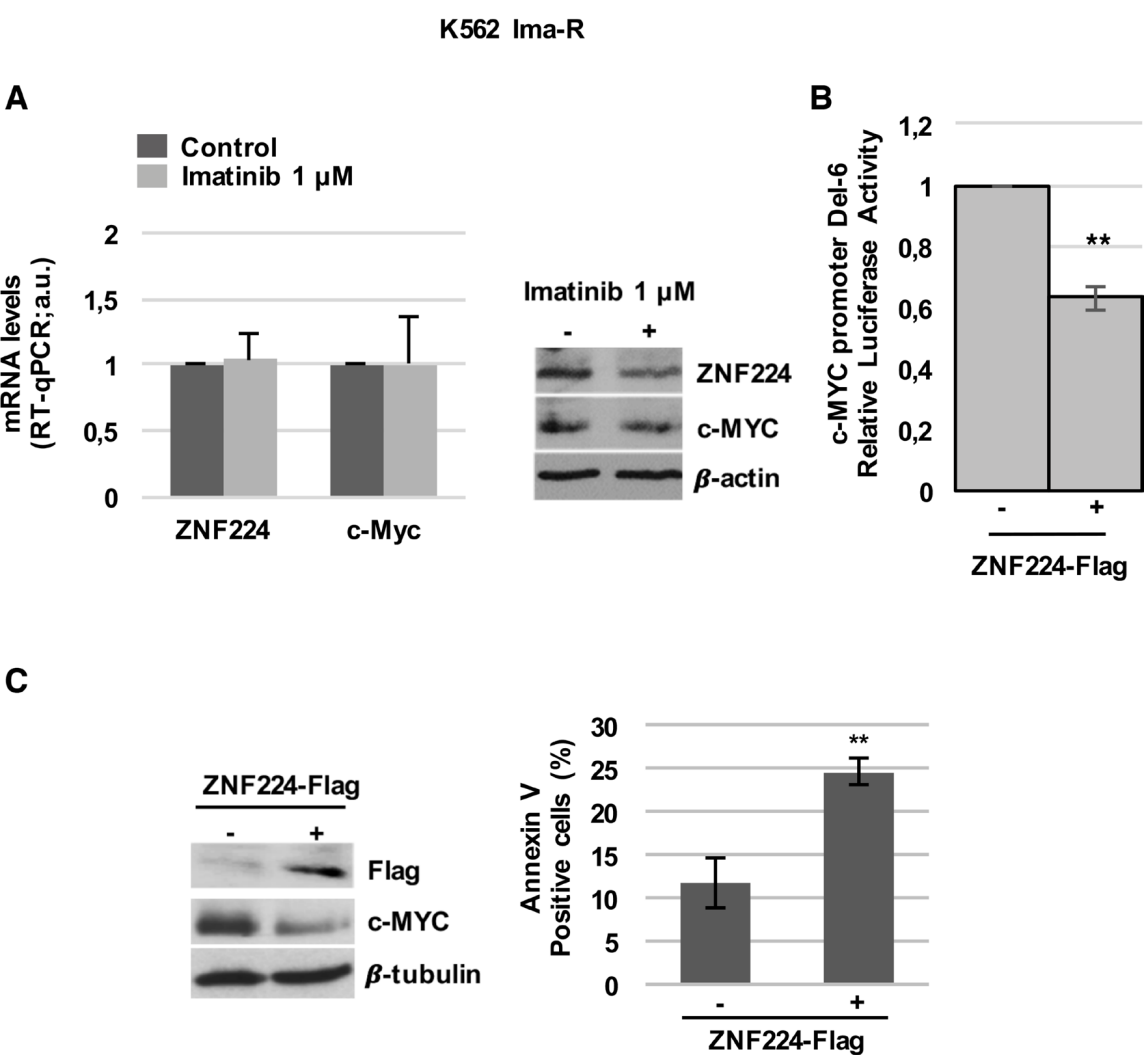
ZNF224 overexpression reduces c-Myc and induces cell death in K562 Ima-R cells (**A**) K562 Ima-R cells were exposed to Imatinib or vehicle only (DMSO) as control (−) for 48 hours. ZNF224 and c-Myc mRNA levels were measured by RT-qPCR. Error bars represent standard deviations of three independent experiments (left panel). ZNF224 and c-Myc protein levels were measured by western blot analysis. β-actin was used as loading control. One representative blot out of two is presented (right panel). (**B**) K562 Ima-R cells were co-transfected with DEL-6 and 3X-Flag ZNF224 or 3X-Flag empty vector as control (−). After 48 h, promoter activity was determined by normalizing Firefly to Renilla luciferase activity. Error bars represent standard deviations of three independent experiments. ^**^p < 0.005. (**C**) K562 Ima-R cells were transfected with 3X-Flag ZNF224 or 3X-Flag empty vector as control (−). c-Myc protein levels were measured by western blot analysis. β-tubulin was used as loading control. One representative blot out of two is presented (left panel). Cell death was determined by annexin V staining followed by flow cytometry. Error bars represent standard deviations of three independent experiments. ^**^p < 0.005.

Prompted by these results, we explored the implication of ZNF224/c-MYC axis in Imatinib resistance. At first, we investigated ZNF224 and c-MYC modulation by Imatinib in K562 cells resistant to Imatinib (K562 Ima-R). In these cells, resistance does not involve neither mutations in Bcr-Abl nor increased Bcr-Abl expression [29]. Interestingly, we observed that Imatinib was not able to induce ZNF224 expression and c-Myc reduction, at both mRNA and protein levels (Figure 5A). As expected, no annexin positivity was observed in K562 Ima-R following Imatinib treatment (Supplementary Figure 2).

It is worth noting that forced expression of ZNF224 in these cells reduced c-Myc promoter activity (Figure 5B), resulting in reduced c-Myc protein levels and a significative increase in cell death (Figure 5C).

### JAK2 inhibitor AG490 reduces c-Myc expression via ZNF224 induction in CML cells

JAK2 pathway plays a pivotal role in c-Myc induction by Bcr-Abl [14–17]. Therefore, since ZNF224 is implicated in the c-Myc repression downstream of Bcr-Abl, we investigated the involvement of ZNF224 in the transcriptional downregulation of c-Myc by AG490, a specific and potent inhibitor of JAK2.

**Figure 6:**
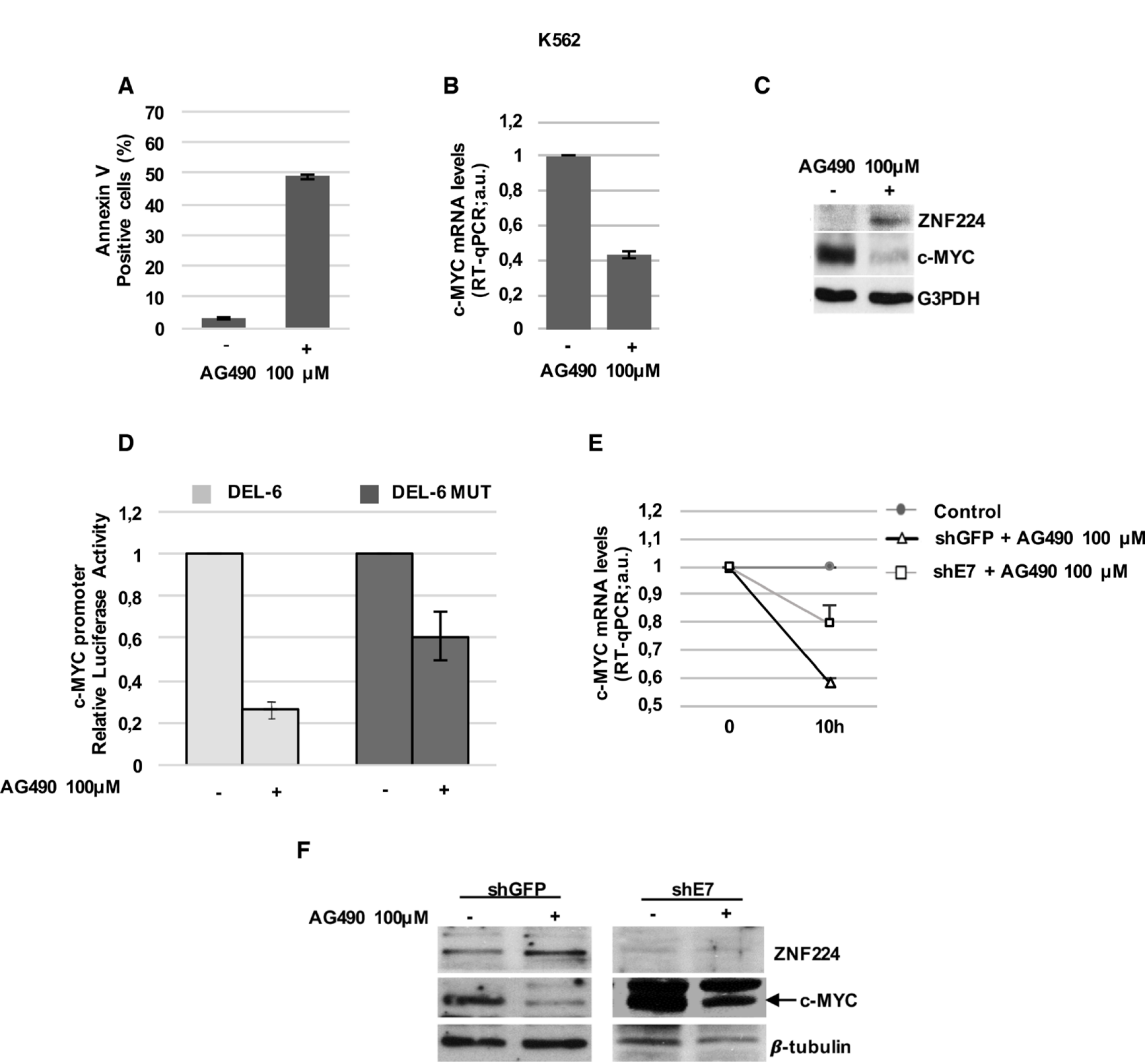
ZNF224 mediates AG490-dependent transcriptional repression on c-Myc (**A**) K562 cells were treated with AG490 for 24h or vehicle only (DMSO) as control (−). Cell death was evaluated by annexin V staining followed by flow cytometry. Results represent the means +/− SD of two independent experiments. (**B**) K562 cells were treated with AG490 for 10 hours or vehicle only (DMSO) as control (−). c-Myc mRNA levels were measured by RT-qPCR. Error bars represent standard deviations of two independent experiments. (**C**) ZNF224 and c-Myc protein levels were measured by western blot analysis. G3PDH was used as loading control. One representative blot out of two performed is shown. (**D**) K562 cells were transiently transfected with DEL-6 or DEL-6-MUT constructs and treated with AG490 or vehicle only (DMSO) as control (−); after 10 h, luciferase activity was determined by normalizing Firefly to Renilla luciferase activity. Error bars represent standard deviations of two independent experiments. (**E**) shE7 and shGFP cells were treated with AG490 or vehicle only (DMSO) as control for 10 hours. c-Myc mRNA levels were measured by RT-qPCR. Relative amounts of c-Myc mRNA levels in shE7 and shGFP cells treated with AG490 were compared to those in shE7 and shGFP treated with DMSO (control). Error bars represent standard deviations of two independent experiments. (**F**) ZNF224 and c-Myc protein levels were measured by western blot analysis. Arrow indicates specific band. β-tubulin was used as loading control. One representative blot out of two is shown.

To investigate these issues, at first we treated K562 cells with AG490 and evaluated ZNF224 and c-Myc expression. As expected, AG490 induced cell death in K562 cells (Figure 6A) and decreased the levels of c-Myc mRNA and protein (Figure 6B and 6C). Interestingly, the reduction of c-Myc was associated with an increase in ZNF224 expression (Figure 6C), thus suggesting the involvement of JAK2 pathway in ZNF224 suppression. Similar results were also obtained in JURL-MK1 CML cells (Supplementary Figure 3). Subsequently, we investigated the effect of AG490 on the activity of the c-Myc promoter constructs, DEL-6 and DEL-6 MUT in K562 cells. We observed that AG490 treatment, similarly to Imatinib (Figure 4B), strongly reduced DEL-6 promoter activity, while exerted a lower repression on DEL-6 MUT (Figure 6D), thus indicating that ZNF224 binding on c-Myc promoter is involved in AG490-dependent repression of the c-Myc gene.

Finally, to definitively demonstrate the involvement of ZNF224 in the c-Myc repression by JAK2 inhibitor, we analyzed the AG490 effect on c-Myc expression in shE7 cells. Interestingly, as shown in Figure 6E and 6F, ZNF224 silencing strongly impaired AG490-mediated downregulation of c-Myc expression at mRNA and protein levels. Taken together, these results indicate that ZNF224 induction mediates, at least in part, AG490 transcriptional repression on c-Myc oncogene in CML cells.

### AG490 induces ZNF224 expression and cell death in Imatinib-resistant CML cells

**Figure 7:**
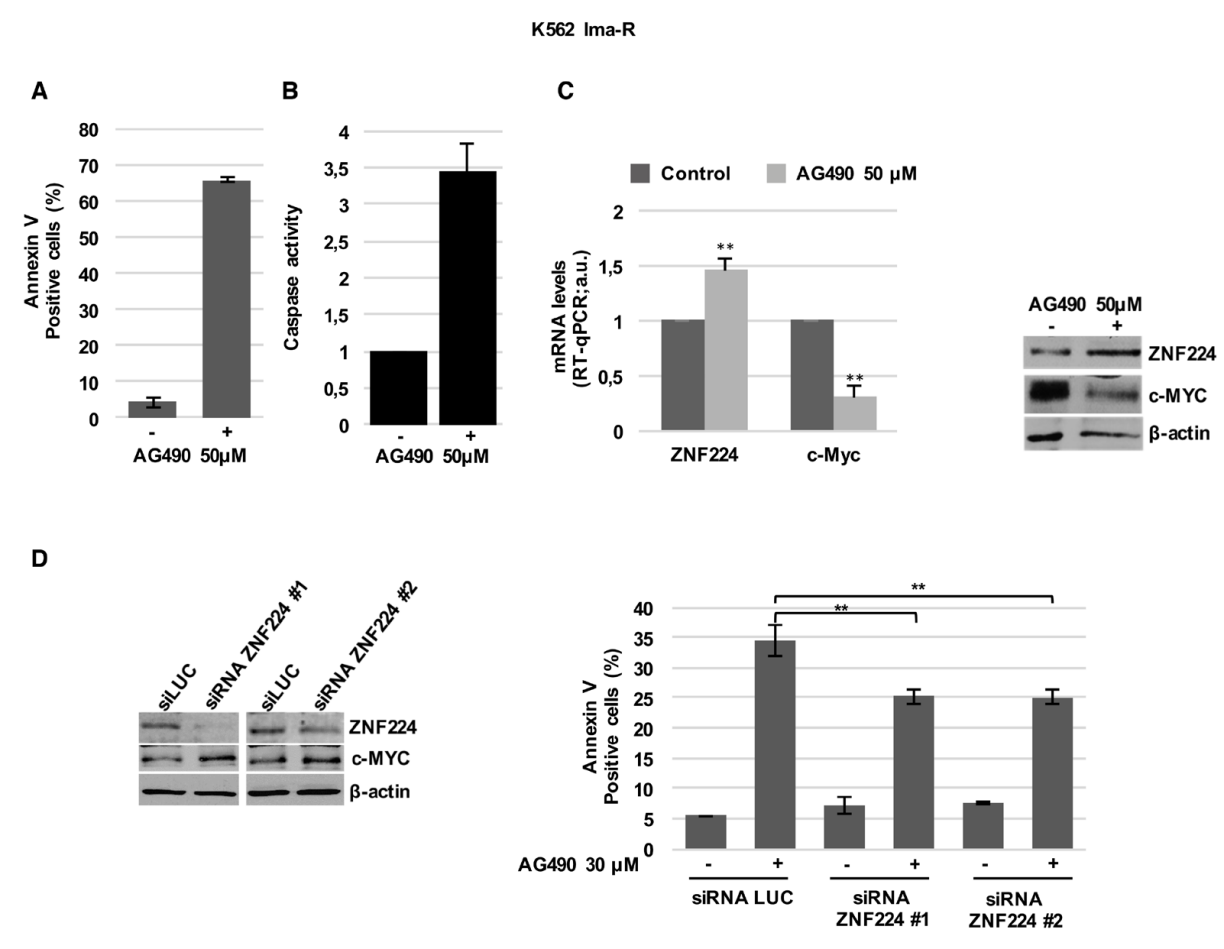
ZNF224 is involved in AG490-induced cell death of K562 Ima-R cells K562 Ima-R cells were exposed to AG490 or vehicle only (DMSO) as control (−) for 48 hours. (**A**) Cell death was determined by annexin V staining followed by flow cytometry. Error bars represent standard deviations of three independent experiments. (**B**) Caspase activity was biochemically measured. (**C**) ZNF224 and c-Myc mRNA levels were measured by RT-qPCR. Error bars represent standard deviations of three independent experiments. ^**^p < 0.005 (left panel). ZNF224 and c-Myc protein levels were measured by western blot analysis. β-actin was used as loading control. One representative blot out of two is presented (right panel). (**D**) K562 Ima-R cells were silenced with two different siRNAs versus ZNF224 (siRNA ZNF224#1 or siRNA ZNF224#2) or with a control siRNA (siLuc) and after 48 hours were collected or exposed to 30 µM AG490 or vehicle only (DMSO) as control (−) for 30 hours. ZNF224 and c-Myc protein levels were evaluated by western blot analysis. β-actin was used as loading control. One representative blot out of two is presented (left panel). Cell death was determined by annexin V staining followed by flow cytometry. Error bars represent standard deviations of three independent experiments. ^**^p < 0.005, (right panel).

Subsequently, we evaluated the effect of AG490 on cell death and ZNF224/c-Myc axis in Imatinib-resistant K562 cells. We first observed that AG490 induces cell death (Figure 7A) and caspase activation (Figure 7B) in K562 Ima-R and similar effects were also found in JURL-MK1 Imatinib-resistant cells (Supplementary Figure 4) [30].

Interestingly, we also observed that the induction of apoptosis in K562 Ima-R cells treated with AG490 was associated with ZNF224 induction and c-MYC reduction (Figure 7C). Similar results were obtained in K562 Nilotinib-resistant cells (K562 Nilo-R) [31], in which AG490 treatment was able to induce apoptosis, an increase of ZNF224 expression and suppression of c-Myc expression (Supplementary Figure 5).

Furthermore, coherently with the role of ZNF224 in repressing c-Myc and inducing cell death in Imatinib-resistant K562 cells, we demonstrated that ZNF224 silencing, with two different siRNAs (siRNA ZNF224 #1 and siRNA ZNF224 #2), increased c-Myc expression and significantly impaired AG490 induced cell death of K562 Ima-R cells (Figure 7D)

## DISCUSSION

Bcr-Abl fusion oncoprotein drives the oncogenic transformation in chronic myelogenous leukemia [2–4] by mainly activating oncogenic pathways [5–9], eventually resulting in the induction of several transcription factors, which strongly sustain oncogenic processes in CML. In this context, the c-Myc transcription factor is necessary for the transforming activity of Bcr-Abl [10].

Bcr-Abl positively regulates c-Myc expression in CML [16]. Consistently, inhibition of Bcr-Abl tyrosine kinase activity by Imatinib, the Tyrosine kinase inhibitor (TKI) used as the frontline drug in CML therapy [32, 33], reduces c-Myc expression and tumoral features of CML cells. A crucial problem linked to TKIs treatment is that the residual leukemic cells accumulate new mutations in Bcr-Abl fusion protein or in other downstream signalling or effector molecules, thus resulting in a refractory response and resistance to TKIs [34]. Several findings showed that elevated c-Myc expression is found in CML blast crisis and correlated with poor response to Imatinib [11]. On the other hand, c-Myc decreased expression represents a key step for Imatinib sensitivity in CML cells [12, 13].

We previously showed that the Kruppel-like zinc-finger protein ZNF224 plays a crucial role in ara-C-induced apoptosis of CML cells, acting as a WT1 transcriptional cofactor [22–24]. Most recently, we demonstrated that ZNF224 expression is down-regulated both in BCR-ABL positive cell lines and in primary CML samples. We also showed that ZNF224 gene expression is negatively regulated by Bcr-Abl oncoprotein via transcriptional repression. In agreement with these findings, we demonstrated that treatment of CML cells with Bcr-Abl inhibitors, such as Imatinib and second generation TKIs increases ZNF224 expression [25].

In this work, we report that ZNF224 binds c-Myc promoter and exerts a transcriptional repression on c-Myc gene in CML, independently of WT1 interaction. In fact, we show that ZNF224 is able to downmodulate c-Myc expression via a transcriptional mechanism in K562 cells, which express high endogenus levels of WT1, as well as in HEK293 cells, which do not express endogenous WT1. Thus, these findings identify the suppression of c-Myc expression as a new mechanism by which ZNF224 operates as a tumor suppressor in CML.

Furthermore, we identify a ZNF224-binding element in the c-Myc promoter that is essential for ZNF224 transcriptional repression on c-Myc in CML cells.

However, since WT1 transcription factor also plays a role in c-Myc transcriptional regulation in CML [35], we cannot exclude that ZNF224 could downmodulate c-Myc also by acting as a WT1 transcriptional cofactor and by suppressing WT1-mediated transactivation of c-Myc gene in CML, as already demonstrated for other WT1 target genes [23, 24].

We also show that ZNF224 repression on c-Myc in CML results in a decreased proliferation and c-Myc proliferative network, leading to a decreased cyclin D1 expression, a positive c-Myc target gene [36], and an increased p21 expression, a negative c-Myc target gene [37–42].

However, it is conceivable that ZNF224 could regulate expression of the above-mentioned genes also by a different mechanism. Indeed, it was recently demonstrated that ZNF224 increases miR-663a transcription, which in turn binds to 3′ UTR of p21 to decrease its expression in MCF-7 breast cancer cell line [43]. Interestingly, miR-663 is a tumour suppressor in CML, which may suppress proliferation in part by enhancing cell apoptosis [44].

Importantly, we show that ZNF224 plays a central role in Imatinib-dependent c-Myc repression in CML. In accordance with the key role of c-Myc reduction in Imatinib responsiveness [12, 13] and with the already described pro-apoptotic mechanisms driven by ZNF224 in CML cells, ZNF224 knockdown led to an impairment of Imatinib-induced cell death.

However, we cannot rule out that ZNF224 induction also plays a role in the Imatinib-mediated proliferative arrest, as already described for THAP11, another important transcriptional repressor of c-Myc oncogene in CML, downstream Bcr-Abl [45].

We also demonstrate that ZNF224 forced expression in Imatinib-resistant K562 cells [27] is accompanied by c-Myc reduction and cell death induction. Altogether, these results highlight the role of ZNF224 in Imatinib responsiveness and suggest that its induction could contribute to circumvent Imatinib resistance in CML.

It has been previously demonstrated by Xie *et al.* [16] that Bcr-Abl-mediated induction of c-Myc expression is dependent on activated JAK2 tyrosine kinase, that plays a key role in the stabilization of c-Myc protein and in the induction of c-Myc mRNA transcription in CML, although the molecular mechanisms of this induction have not been clarified.

Consistently, JAK2 kinase inhibitors, such as AG490, strongly reduce c-Myc expression in CML cells and induce apoptosis [14, 16], thus representing promising molecular tools to bypass Imatinib resistance in CML [17–19].

By exploiting the newly discovered link between ZNF224 and c-Myc downstream of Bcr-Abl in CML, we show that ZNF224 expression is induced by AG490 in both sensitive and imatinib-resistant K562 cells. Interestingly, we also demonstrated that AG490 is able to induce apoptosis at least in part via ZNF224 induction and consequent c-Myc repression. These findings strongly suggest that the induction of ZNF224 expression, by targeting signaling pathways downstream of Bcr-Abl, may be exploited as basis for the development of new therapeutic approach in imatinib-resistant CML.

## MATERIALS AND METHODS

### Cell lines and reagents

HEK293T human cell line was cultured in Dulbecco’s modified Eagle’s medium (Sigma-Aldrich, St Louis, MO, USA) supplemented with 10% fetal calf serum and 100 µg/ml streptomycin-penicillin mix (Sigma-Aldrich) at 37°C in 5% CO2. K562 and JURL-MK1 human cell lines were cultured in RPMI 1640 (Sigma-Aldrich) supplemented with 10% fetal calf serum and 100 µg/ml penicillin-streptomycin mix (Sigma-Aldrich) at 37°C in 5% CO2. K562 and JURL-MK1 cells were treated with 1 μM Imatinib (Novartis Pharma, Basel, Switzerland), 20 nM of Nilotinib (Novartis Pharma), 10, 30, 50 or 100 μM AG490 (Sigma-Aldrich), 500ng/ml Puromycin (Sigma-Aldrich). K562 Ima-R clones were established and described in [29]. K562 Nilo-R clones were established as described in [31]. JURL-MK1 Ima-R clones were established as described in [30].

### RNA isolation, reverse transcription and real-time qPCR

Total RNA was isolated using the Quick-RNA™ MiniPrep Plus (Zymo research, Irvine, CA, USA) according to the manufacturer’s protocol. 1 μg of RNA was reverse-transcribed using the iScript Reverse Transcription Supermix for RT-qPCR (Bio-Rad, Berkeley, CA, USA), as recommended by the manufacturer. Real-time PCR was carried out in a Real-Time CFX 69 System (Bio-Rad) using the Master Mix SYBR Green (Bio-Rad). For RT-qPCR analysis of mRNA levels, we used specific primers for ZNF224 (Fw 5′-GGGCTGTCTTGGCACAATTC-3′; Rev 5′-TTGCCTCCTTGAACGTGGTC-3′) and c-Myc (Fw 5′-ACTCTGAGGAGGAACAAGAA-3′; Rev 5′- TGGAGACGTGGCACCTCTT-3′). Abl (Fw 5′-GATGTAGTTGCTTGGGACCCA-3′; Rev 5′- TGGAGATAACACTCTAAGCATACT-3′) and b2 microglobulin (Fw 5′- CCGTGGCCTTAGCTGTGCT-3′; Rev 5′- TCGGATGGATGAAACCCAGA-3′) specific primers were used as control. The relative quantification in gene expression was determined using the ΔΔCT method.

### Cell lysates and western blot assays

Total cell lysates were prepared by homogenization in modified RIPA buffer as previously described [46]. Western blot membranes were incubated with the following antibodies: anti-ZNF224 (rabbit polyclonal, T3) [47] diluted 1:300 in Super-Block Blocking Buffer (Thermo Fisher Scientific, Waltham, MA, USA), anti-c-Myc, anti-Cyclin D1, anti-p21, anti-p27 and anti-PCNA (Santa Cruz Biotechnology, CA, USA) diluted 1:500, anti-GAPDH (Santa Cruz Biotechnology) diluted 1:1000, anti-β-actin (Sigma-Aldrich) diluted 1:1000, anti-Flag and anti-β-Tubulin (Upstate, Lake Placid, NY) diluted 1:1000, anti-caspase 3 cleaved (Santa Cruz Biotechnology). Signals were detected with ImmunoCruz Western Blotting Luminol Reagent (Santa Cruz Biotechnology).

The band intensity of c-Myc was quantified by densitometry, using imageJ software. The expression levels of c-Myc in cells transfected with 0,5 µg of 3X-Flag ZNF224 was arbitrarily set to 1.

### Transient and stable transfection

HEK293 cells were transiently transfected using Metafectene (Biontex, Munchen, Germany) with either 0,5 μg or 1 μg or 2 μg of 3X-Flag ZNF224 expression plasmid. As control, 0,5 μg or 1 μg or 2 μg of 3X-Flag empty vector were transfected. K562 cell lines were transiently transfected, using Lipofectamine 2000 (Thermo Fisher), with either 1,5 μg of 3X-Flag ZNF224 expression plasmid or 1,5 μg of two different shRNA specific for ZNF224 (shC3 and shE7). As control, 1,5 μg of 3X-Flag empty vector or shRNA specific for GFP were transfected.

To obtain K562 cells stably knocked-down for ZNF224, K562 cells transfected with two different ZNF224 shRNAs (shC3 and shE7) or with an shRNA targeting GFP (shGFP) as control were cultured in RPMI supplemented with 10% FBS and selected with 500 ng/mL Puromycin (Sigma-Aldrich) for one week.

K562 Ima-R cells were transiently transfected, using Lipofectamine RNAiMAX Reagent (Thermo Fisher) with either 90 pmol of two different pool of siRNAs specific for ZNF224: siRNA ZNF224 pool #1 (ZNF224HSS144559, by Thermo Fisher) or siRNA ZNF224 pool #2 (ZNF224HSS144560, by Thermo Fisher). As control, 90 pmol of siRNA specific for Luciferase were transfected. 48 h after transfection, cells were collected or treated.

### Luciferase reporter assays

HEK293 cells were transiently transfected with luciferase reporter plasmids containing the c-Myc promoter (0,2 μg of c-Myc Del-2/Del-3/Del-6 plasmids) and pRL-CMV plasmid coding for the renilla luciferase to normalize (1:10 the ratio between renilla and c-Myc construct) using Metafectene (Biontex). c-Myc Del-2 and Del-3 plasmids were a gift from Bert Vogelstein (Addgene plasmid # 16603) [48]. c-Myc Del-6 plasmid was a gift from Joan Massague (Addgene plasmid # 14969) [48]. HEK293 cells were co-transfected with 0,2 μg, 0,4 μg or 0,6 μg of 3X-Flag ZNF224 expression plasmid or with 0,2 µg, 0,4 µg, 0,6 µg of 3X-Flag empty vector as control (–).

K562 and K562 Ima-R cell lines were transiently transfected with 0,2 μg of c-Myc Del-6 luciferase reporter plasmid and pRL-CMV plasmid to normalize, using Lipofectamine 2000 (Thermo Fisher). K562 and K562 Ima-R cell lines were co-transfected with 1 μg of 3X-Flag ZNF224 expression plasmid or with 1 μg of 3X-Flag empty vector, as control. After 24 or 48 h, luciferase activity was measured in HEK293, K562 or K562 Ima-R cell lines, using the Dual-Luciferase Reporter Assay System (Promega Corporation, WI, USA), according to the manufacturer’s instructions.

### Chromatin immunoprecipitation assay

Cross-linked chromatin was prepared from HEK293 or K562 cells and immunoprecipitated with anti-ZNF224 antibody (Santa Cruz Biotechnology), as previously described [49]. Immunoprecipitated DNA was then analyzed by quantitative RT-qPCR using a Master Mix SYBR Green (Bio-Rad) and specific primers for: c-Myc promoter (P2 region) (Fw: 5′-TCGGGGCTTTATCTAACTCG-3′; Rev: 5′-GCTGCTATGGGCAAAGTTTC-3′) and Unrelated c-Myc region [Fw 5′-GAAGCGGAAATTGCAGTGAG-3′; Rev:5′-AGGGATAGGGTCTTGCTACG-3′). The percentage of DNA immunoprecipitated with anti-ZNF224 antibody was calculated relative to the ChIP input DNA.

### Cell counting and annexin V assay

For cell number determination, K562 cells stably silenced for ZNF224 were plated at a density of 1 × 10^4^ cells/mL in a 24 well plate and viable cells were counted from 1 to 4 days by trypan blue exclusion every 24 hours. For cell doubling time determination, K562 cells stably silenced for ZNF224 were plated at a density of 4 × 10^4^ cells/mL in a 12 well plate and viable cells were counted every 24 hours from plating. Cell doubling time was calculated by Log_2_ (n° counted cells/n° plated cells).

For annexin V staining, 1 × 10^5^ cells were washed twice in cold PBS, then resuspended in cold annexin V-binding buffer (5 M NaCl, 1 M CaCl2, 1M HEPES buffer/NaOH pH 7.4) and stained with annexin V-APC (BD Biosciences, San Jose, CA, USA). After incubation in the dark on a shaker for 15 min at 4°C, cells were analyzed on FACS flow cytometer BD Biosciences Accuri C6 Flow Cytometer (BD Biosciences).

### Site-directed mutagenesis of c-Myc promoter reporter construct

Mutant constructs were generated by the QuikChange^®^ site-directed mutagenesis kit (Stratagene, San Diego, CA, USA) using c-Myc Del-6 (–109 to +334) as DNA template. Mutagenesis was performed in according to the manufacturer’s instructions. An oligonucleotide to convert AGA to GCG in c-Myc promoter, was synthesized (5′ - GTGGAAGAGCCGGGCGAGCGCGGCTGCGCTGCGGGCGTCC -3′ N bp = 40; %GC = 80; %MM = 7.5; Tm = 89.925°C). Obtained mutation of the Del-6 construct was confirmed by DNA sequencing.

### BrdU incorporation assay

Cell proliferation measured with 5-bromo-20-deoxy-uridine labelling was performed with a Detection Kit II (Roche Diagnostics Corporation, Indianapolis, IN, USA) following the manufacturer’s instructions. Briefly, K562 cells stable silenced for ZNF224 were plated at a density of 3 × 10^5^ cells/ 6-well plate. To obtain ZNF224 overexpression, 4 × 10^5^ K562 cells were transiently transfected with 7 µg of 3X-Flag ZNF224 expression plasmid or 3X-Flag empty vector for 24 hours, and then 2 × 10^5^ of transfected cells were plated into 6-well plates. K562 cells knocked-down or ovexpressing ZNF224 were incubated in the presence of 10 μM BrdU for 3 or 5 hours, then collected, fixed with ethanol and incubated with anti-BrdU monoclonal antibody (Roche). The percentage of BrdU incorporation was measured on FACS flow cytometer (BD Biosciences Accuri C6 Flow Cytometer).

### Caspase activity measurement

K562 cells were lysed in lysis buffer for 30 min at 4°C [50]. The lysates were cleared at 10,000 *g* for 15 min at 4°C. Each assay was performed using 25 μg of protein extract incubated in a 96-well plate with Ac-DEVD-AMC (7-amino-4-methylcoumarin) or Ac-LEHD-AMC (0,2 mmol/L) for various times at 37°C, as described [50].

### Statistical analysis

All data are presented as mean±SD. Statistical analysis was performed with Student’s *t*-test [two-tailed]. ^*^p < 0.05 , ^**^p < 0.005. *P* value < 0.05 was defined as statistically significant.

## SUPPLEMENTARY MATERIALS FIGURES



## Abbreviations

CML: Chronic myelogenous leukemia
WT1: Wilms Tumor protein 1
TKIs: tyrosine kinase inhibitors
TSS: transcriptions start sites
ChIP: Chromatin immunoprecipitation assays
