# Supplementary material for: Role of ZNF224 in c-Myc repression and imatinib responsiveness in chronic myeloid leukemia
Source: Oncotarget. 2017 Dec 15;9(3):3417–31. doi: 10.18632/oncotarget.23283 (PMC5790473; doi:10.18632/oncotarget.23283)
Supplement: Supplementary file 1 [file oncotarget-09-3417-s001.pdf]

Role of ZNF224 in c-Myc repression and imatinib responsiveness in chronic myeloid leukemia

SUPPLEMENTARY MATERIALS

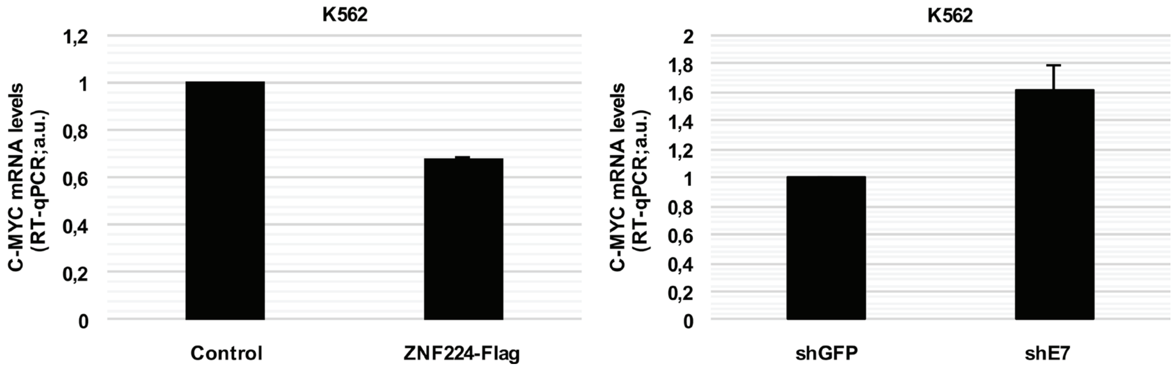

Supplementary Figure 1: ZNF224 expression affects c-Myc mRNA levels. ZNF224 and c-Myc mRNA levels were measured by RT-qPCR in K562 transfected with 3X-Flag ZNF224 or 3X-Flag empty vector as control (left panel). ZNF224 and c-Myc mRNA levels were measured by RT-qPCR in shE7 and shGFP K562 cells (right panel). Error bars represent standard deviations of two independent experiments.

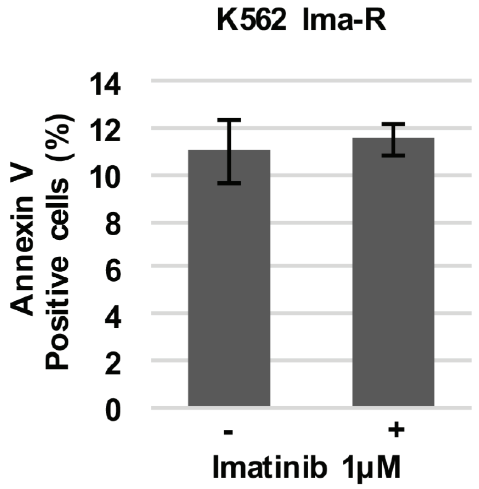

Supplementary Figure 2: Imatinib does not induce cell death in K562 Ima-R cells. K562 Ima-R cells were exposed to 1 µM Imatinib or vehicle only (DMSO) as control (–) for 48 hours and cell death was determined by annexin V staining followed by flow cytometry. Error bars represent standard deviations of two independent experiments.

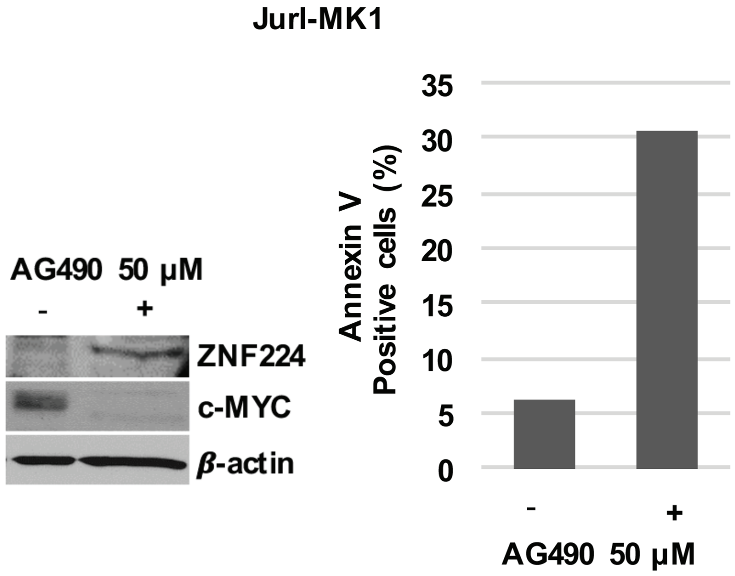

Supplementary Figure 3: AG490 increases ZNF224 expression and induces cell death in Jurl-MK1 CML cells. Jurl-MK1 cells were exposed to 50 μM AG490 for 48 hours or vehicle only (DMSO) as control (–). ZNF224 and c-Myc protein levels were measured by Western Blot analysis. ß-actin was used as loading control. Cell death was evaluated by annexin V staining followed by flow cytometry.

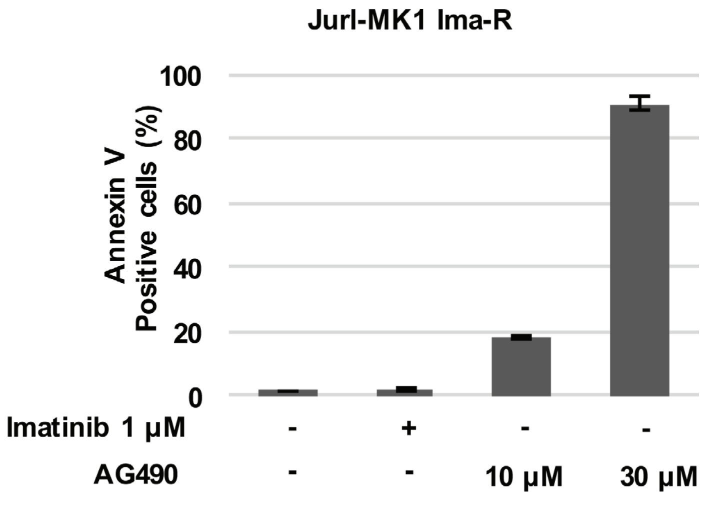

Supplementary Figure 4: AG490 induces cell death in Jurl-MK1 Ima-R cells. Jurl-MK1 Ima-R cells were exposed to 1 μM Imatinib or increasing concentrations of AG490 (10 μM and 30 μM) or vehicle only (DMSO) as control (–) for 48 hours. Cell death was determined by annexin V staining followed by flow cytometry. Error bars represent standard deviations of two independent experiments.

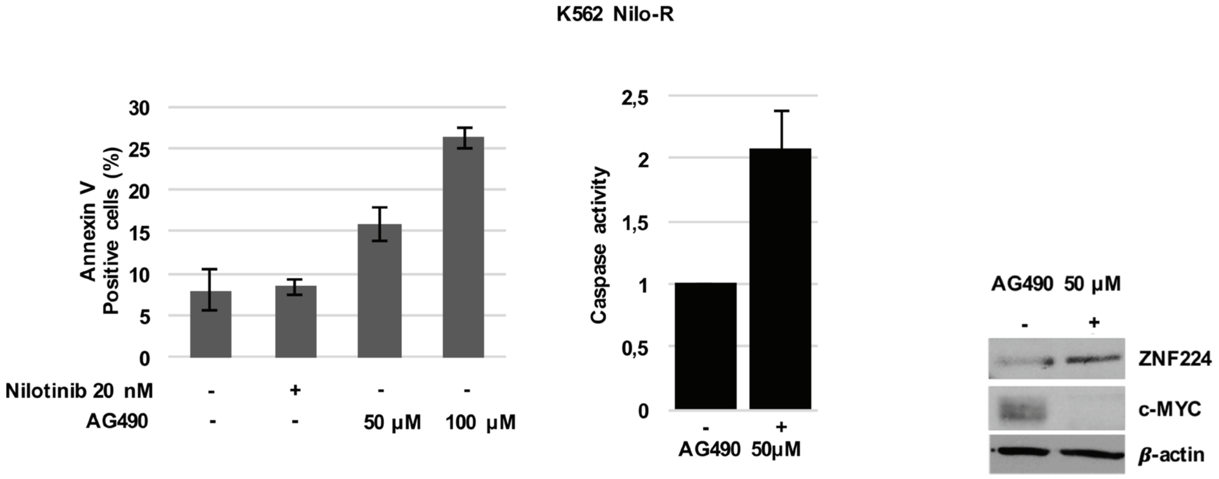

Supplementary Figure 5: AG490 induces cell death and increases ZNF224 expression in K562 Nilo-R cells. K562 Nilo-R cells were exposed to 20 nM Nilotinib or increasing concentrations of AG490 (50 µM and 100 µM) or vehicle only (DMSO) as control (–) for 48 hours. Cell death was determined by annexin V staining followed by flow cytometry. Error bars represent standard deviations of two independent experiments. Caspase activity was biochemically measured. Error bars represent standard deviations of two independent experiments. ZNF224 and c-Myc protein levels were measured by Western blot analysis. ß-actin was used as loading control. One representative blot out of two is presented.
